# Autoantibodies to aberrantly glycosylated MUC1 in early stage breast cancer are associated with a better prognosis

**DOI:** 10.1186/bcr2841

**Published:** 2011-03-08

**Authors:** Ola Blixt, Deanna Bueti, Brian Burford, Diane Allen, Sylvain Julien, Michael Hollingsworth, Alex Gammerman, Ian Fentiman, Joyce Taylor-Papadimitriou, Joy M Burchell

**Affiliations:** 1 Copenhagen Center for Glycomics (CCG), Departments of Cellular and Molecular Medicine and Dentistry, Faculty of Health Sciences, University of Copenhagen, Blegdamsvej 3, DK-2200 Copenhagen N, Denmark; 2 Breast Cancer Biology, Research Oncology, King's College London, 3rdFloor Bermondsey Wing, Guy's Hospital, London SE1 9RT UK; 3 Computer Learning Research Centre, Royal Holloway, University of London, Egham Hill, Egham, TW20 0EX, UK; 4 Current address: Breast Cancer Biology, Research Oncology, King's College London, 3rd Floor Bermondsey Wing, Great Maze Pond, Guy's Hospital, London SE1 9RT, UK; 5 Research Oncology, King's College London, 3rd Floor Bermondsey Wing, Great Maze Pond, Guy's Hospital, London SE1 9RT, UK; 6 University of Nebraska Medical Center, Eppley Institute for Research in Cancer and Allied Diseases, 986805 Nebraska Medical Center Omaha, NE 68198-6805, USA

## Abstract

**Introduction:**

Detection of serum biomarkers for early diagnosis of breast cancer remains an important goal. Changes in the structure of O-linked glycans occur in all breast cancers resulting in the expression of glycoproteins that are antigenically distinct. Indeed, the serum assay widely used for monitoring disease progression in breast cancer (CA15.3), detects a glycoprotein (MUC1), but elevated levels of the antigen cannot be detected in early stage patients. However, since the immune system acts to amplify the antigenic signal, antibodies can be detected in sera long before the antigen. We have exploited the change in O-glycosylation to measure autoantibody responses to cancer-associated glycoforms of MUC1 in sera from early stage breast cancer patients.

**Methods:**

We used a microarray platform of 60mer MUC1 glycopeptides, to confirm the presence of autoantibodies to cancer associated glycoforms of MUC1 in a proportion of early breast cancer patients (54/198). Five positive sera were selected for detailed definition of the reactive epitopes using on chip glycosylation technology and a panel of glycopeptides based on a single MUC1 tandem repeat carrying specific glycans at specific sites. Based on these results, larger amounts of an extended repertoire of defined MUC1 glycopeptides were synthesised, printed on microarrays, and screened with sera from a large cohort of breast cancer patients (*n *= 395), patients with benign breast disease (*n *= 108) and healthy controls (*n *= 99). All sera were collected in the 1970s and 1980s and complete clinical follow-up of breast cancer patients is available.

**Results:**

The presence and level of autoantibodies was significantly higher in the sera from cancer patients compared with the controls, and a highly significant correlation with age was observed. High levels of a subset of autoantibodies to the core3MUC1 (GlcNAcβ1-3GalNAc-MUC1) and STnMUC1 (NeuAcα2,6GalNAc-MUC1) glycoforms were significantly associated with reduced incidence and increased time to metastasis.

**Conclusions:**

Autoantibodies to specific cancer associated glycoforms of MUC1 are found more frequently and at higher levels in early stage breast cancer patients than in women with benign breast disease or healthy women. Association of strong antibody response with reduced rate and delay in metastases suggests that autoantibodies can affect disease progression.

## Introduction

Studies investigating the role of cancer associated antigens in the diagnosis of malignancy, and in cancer progression have focused on the MUC1 antigen, which is expressed in an aberrantly glycosylated form in most carcinomas including breast cancer [1-4]. Assays to detect the cancer-associated antigen in serum (for example, the CA15.3 assay) are widely used for monitoring disease progression and response to therapy in some late stage breast cancer patients, but this assay does not detect elevated levels of MUC1 in serum from patients with early stage disease [5]. The detection of biomarkers in serum that could diagnose the presence of breast cancer, therefore, remains an important goal.

In contrast to antigens, autoantibodies can be detected in serum from early stage patients, the immune system serving to amplify the detection of antigen [6-10]. Most of the studies to date analyzing the profile of antibodies to MUC1 have used ELISA assays, generally detecting all Ig antibodies to the unglycosylated form of the mucin, or undefined glycoforms [1-3,11,12]. We have developed a novel O-glycopeptide array-based assay detecting IgG autoantibodies to specific glycoforms of MUC1 [13], which has low backgrounds and detects autoantibodies in sera from cancer patients. Our approach utilizes the added dimension of the changes in O-glycosylation that occur in cancer. This is in contrast to other methods used to identify specific targets for autoantibodies that do not allow for the detection of antibodies to epitopes resulting from cancer-associated post-translational modifications [1,10,14].

The pattern of O-glycosylation of membrane or secreted glycoproteins is consistently altered in virtually all cancers. In this type of glycosylation a family of polypeptide GalNAc-transferases add the sugar N-acetylgalactosamine (GalNAc) to serine and threonine residues in the core protein, and the glycan chain is extended by adding other sugars, individually and sequentially in the Golgi pathway. Chain termination occurs by adding specific sugars such as sialic acid or fucose [15]. In breast cancer, glycan chains can be terminated early, in part due to changes in the expression of some glycosyltransferases [16], resulting in the expression of novel glycopeptide epitopes, which alter the immunogenicity of the glycoprotein [17,18]. In the case of mucin glycoproteins, because of the presence of multiple tandem repeats which are rich in serine and threonine, the protein carries hundreds or even thousands of O-glycans, and changes in their composition can dramatically affect the immunogenicity of the cancer-associated glycoform.

**Figure 1:**
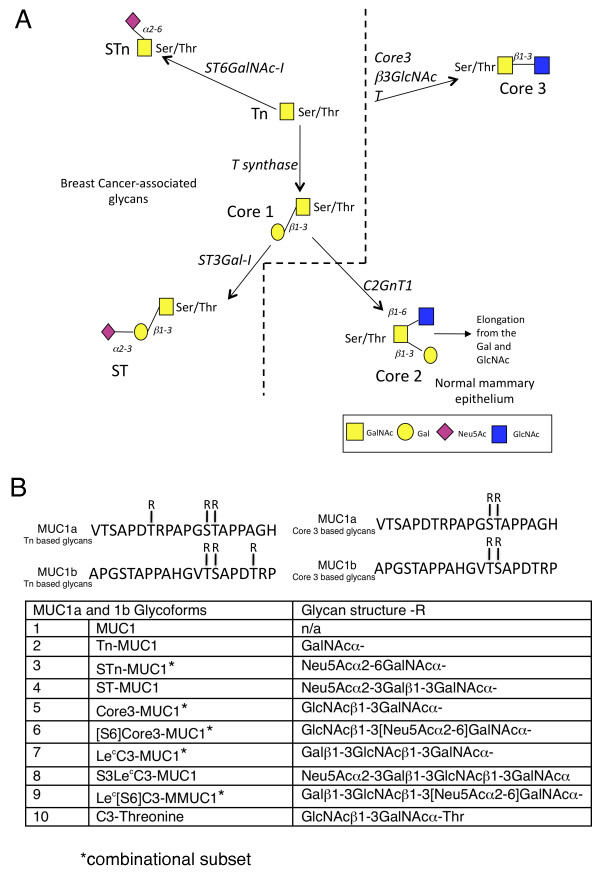
**O-linked glycosylation in breast cancer and normal mammary gland and informative glycopeptides used**. **A**, aberrant O-linked glycosylation results in the expression of Tn, STn, Core 1 and ST glycoforms, left of the dotted line. O-glycans in the normal mammary gland are mostly core2 based, right bottom. Note: core3 based glycans (top right) have not been demonstrated in the normal or malignant breast. **B**, Sequence of MUC1a and MUC1b showing sites of glycosylation and glycans carried.

MUC1 is the most widely expressed of the mucins, being found to some degree in epithelia lining glands or ducts. It is, however, upregulated in breast and other carcinomas as well as being differently glycosylated [16]. The tandem repeat sequence in MUC1 is composed of 20 amino acids (V**TS**APD**T**RPAPG**ST**APPAHG) within which there are five potential O-linked glycosylation sites (serines and threonines), and the number of tandem repeats varies between 25 and 125 depending on the allele. The truncated glycans carried on breast cancer-associated MUC1 include the single sugar, αGalNAc (Tn), its sialylated derivative (STn), T (Galβ1,3GalNAcα) and sialylated T (see Figure 1). Our initial pilot studies used an array of 60mer MUC1 glycopeptides carrying various truncated glycans (enzymatically synthesized on the peptide in solution) for the detection of autoantibodies in a small number of breast cancer patients [13]. While antibodies to both Tn and STn MUC1 glycopeptides were detected in some patients, a more frequent antibody response was seen to MUC1 carrying a truncated core3 glycan (GlcNAcβ1-3GalNAcα).

We have recently demonstrated a novel method of on-chip synthesis of 20mer MUC1 glycoproteins to explore in detail the positions of the O-glycans attached to the MUC1 peptide in the glycopeptides eliciting an autoantibody response [19]. Based on the initial analysis, and work presented here using on-chip synthesis, corresponding glycopeptides and additional glycoforms were selected for preparative scale synthesis in order to analyze antibody responses in a large cohort (395 serum samples) of breast cancer patients with Stage I or Stage II disease, comparing the autoantibody response to that in sera from patients with benign disease or age-matched healthy females. As with the pilot study, we find that the dominant antibody responses were to the MUC1 carrying the truncated core3 and STn glycans.

The analysis of autoantibodies to MUC1 glycoforms demonstrated an autoantibody response in early stage breast cancer patients that is highly significantly related to age, emphasizing the importance of using age matched controls in screening. Moreover, autoantibodies to specifc glycoforms of MUC1 were detected more frequently and at a higher level in breast cancer patients than in patients with benign breast disease or healthy females. Interestingly, autoantibody induction to the core3 based and STn glycoforms of MUC1 is significantly related to a lower incidence of metastases and increased time to metastasis, suggesting that autoantibodies to cancer-associated glycoforms of MUC1 may play a role in the development and progression of the disease.

## Materials and methods

### Construction and quality control of glycopeptide micro-arrays

#### 

##### Microarrays with glycopeptides extended from Tn MUC1 glycopeptides on-chip used for epitope mapping

**Figure 2:**
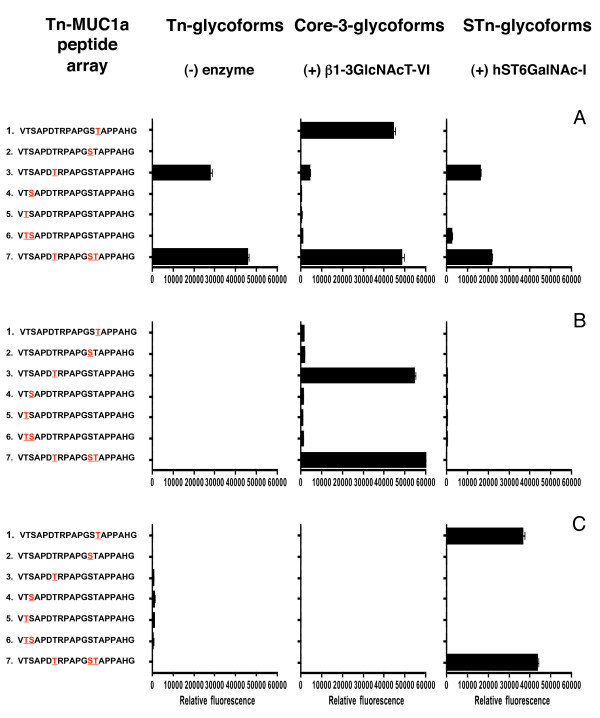
**Epitope mapping of autoantibodies in sera from breast cancer patients**. Arrays were produced by on-slide glycosylation of the MUC1a glycopeptides carrying Tn at the sites indicated in red using core3 β3GlcNAc-T6 (middle panel) and ST6GalNAc-I (right panel). The arrays were stained with diluted sera (1:20) and Cy3 labelled anti-human-IgG. **A, B, C **show data obtained with sera from three individual patients.

Thirty-one Tn glycopeptides (each carrying one to five glycans) were chemically synthesized and extended on-chip as described (see Figure 2, [19]), using ST6GalNAcI for STn synthesis and the core3 β1-3 GlcNAc transferase for the synthesis of core3MUC1 glycopeptides. Five breast cancer sera selected as having antibodies to core3 MUC1 and/or STn MUC1, on the 60mer arrays, were screened on these arrays of MUC1 monomer glycopeptides.

##### Arrays for screening of the large cohorts of breast cancer patients (*n *= 395), patients with benign breast disease (*n *= 108) and healthy women (*n *= 99)

The Tn-MUC1a, Tn-MUC1b, Core3-MUC1a and Core3-MUC1b glycopeptides were generously provided by GlycoZym Inc. (Beverly, MA, USA). and further elongated enzymatically in solution. Briefly, general enzymatic glycosylation reactions (2 to 5 μg enzyme/100 μg glycopeptide) were performed with one or several recombinant glycosyltransferases, T-synthase dC1GalT1 *Drosophila melanogaster *[17], β3Gal-TV [20], ST3GalI [20] and ST6GalNAcI [17] together with the corresponding donor sugar nucleotides (5 mM) in sodium cacodylate buffer solution (2.0 mL, 0.5 M, pH 7.2) containing manganese(II)chloride and incubated at room temperature for 24 hrs. All glycopeptides synthesised in solution, were individually diluted with water (1 mL), and slowly passed through a C-18 Isolute cartridge (0.5 g, previously washed with 5 mL of MeOH followed by 20 mL of water). After washing the column with water (10 mL), the desired material was eluted with a stepwise gradient (0 to 90% MeOH:water (0.5 mL aliquots). All fractions were analyzed by MALDI-TOF and fractions containing the desired product were pooled and lyophilized. Recombinant MUC1 based glycoproteins carrying ST and T were produced in CHOK1 cells as described by Bäckström *et al. *[21] and those without O-linked glycans or carrying the Tn glycan were produced in CHO ldlD cells. Glycopeptides and glycoproteins were used for printing of 48-well slides (for list of glycopeptides, see Table S1 in Additional file 1).

### Printing of slides

Printing was performed on Schott Nexterion^® ^Slide H, Schott Nexterion^®^. Slide H MPX16 or MPX48 (Schott AG, Mainz, Germany). All salts for all the buffers, including TES, Triton-X-100 Tween 20, and ethanolamine were from Merck (Whitehouse Station, NJ, USA). Printing of the 48-well microarray slides was performed using a BioRobotics MicroGrid II spotter (Genomics Solution, Huntingdon, Cambridgeshire, UK) using Stealth 3B Micro Spotting Pins (Telechem International ArrayIt Division, Sunnyvale, CA, USA). The compounds were distributed into 384-well source plates (BD Falcon Microtest™ from BD Biosciences, Le Pont De Claix, France), 20 μL per well at 100 μM final concentration diluted in print buffer (PB) (150 mM phosphate, 0.005% CHAPS pH 8.5). The MPX16 well slides were printed in quadruplicates using an 8-pin (8 × 2) configuration within a 28 × 28 subgrid at a 0.20 mm pitch between each spot, and the MPX48 well slides were printed in three replicates using an 24-pin (6 × 4) configuration within a 12 × 12 subgrid at a 0.15 mm pitch between each spot. The pin dwell time in the wells was four seconds and the pins underwent three wash cycles in between source plate visits. The complete 8 × 2 or 6 × 4 array pattern was printed on a 16-well or 48-well slide in duplicate respectively. Scanning of the slides was performed on ProScanArray HT Microarray Scanner (PerkinElmer, Cambridge, Cambridgeshire, UK) followed by image analysis with ProScanArray Express 4.0 software (PerkinElmer). Human IgG was spotted (100 ug/mL in PB) for control of second antibody reactivity and for orientation of the scanned slides. Data were analyzed and plotted using Microsoft^® ^Excel, GraphPad Prism software or MathWorks Matlab software (Microsoft^®^, Reading, Berkshire, UK). Quality control of printed glycopeptides were visualized by staining with glycoform-specific lectins and antibodies as described [13,19] (see Figure S1 in Additional file 2).

### Sera collection

The sera from cancer patients used in this study are part of a series of consecutive Stage I and Stage II breast cancer patients collected at Guy's Hospital, London between 1975 and 1980, known as the P-series. A total of 400 sera were randomly selected of which 5 did not fit the criteria and were, therefore, excluded leaving a cohort of 395. Complete clinical follow-up is available for these patients. The mean age of the cancer patients at the time of collection (one day pre-surgery) was 59.9 years, see Table 1 for clinical and treatment parameters of this cohort. Sera were taken with informed patient consent for the study of prognostic markers and were not taken as part of a particular clinical trial. At the time of taking these samples written informed consent was not required nor was ethnicity recorded. Ethical approval for use of the samples in the present study was obtained from the Outer South East London Research Ethics Committee, REC reference 07/H0809/51. Sera were also collected from 108 patients with benign breast disease who attended Guy's Hospital during the same period. Benign breast disease ranged from breast pain to benign tumours such as fibroadenomas. The mean age of the benign patients at the time of sera collection was 45 years. Sera from 99 healthy volunteer females living on the island of Guernsey formed the normal cohort (mean age 54.7) [22]. Sera were taken from these women between 1977 and 1985. The women who donated these control sera were followed-up for between 25 and 30 years and at the time of sera selection (end of 2008) all except three were still alive and cancer free. However, none of the three that died had been diagnosed with cancer and one died in 2003 aged 102 and two in 2000 aged 93 and 95. Written informed consent had been obtained from each volunteer. This consent covered use of the serum for the investigations into the discovery of cancer biomarkers and access to the womens' medical records. Ethical approval to allow the access to patients' medical records of the volunteers who donated sera to the Guernsey bank has been obtained from the Guernsey and Alderney Committee.

**Table 1:** Distribution of clinical, treatment and outcome parameters of 395 early breast cancer patients

Parameters	**Early stage breast cancer, total number cases = 395**. n (%)
Age at diagnosis	
<50	63 (16)
50 to 59	132 (34)
60 to 69	136 (34)
70+	64 (16)
Stage	
Node negative	200 (51)
Node positive	193 (49)
nk	2
Positive nodes	
0	200 (51)
1 to 3	124 (32)
4+	69 (17)
nk	2
Clin tumour size	
0 to 2 cm	133 (35)
2.1 to 4 cm	176 (46)
>4 cm	74 (19)
nk	16
Tumour type	
Infiltrating ductal	338 (88)
Infiltrating lobular	34 (9)
Other	10 (3)
nk	13
ER Status	
ER negative	131 (38)
ER positive	211 (62)
nk	53
Parity	
0 children	87 (25)
1 to 2 children	171 (48)
3+ children	96 (27)
nk	41
Type of surgery	
Radical mastectomy	372 (94)
Simple mastectomy	16 (4)
Wide local excision	6 (2)
nk	1
Adjuvant radiotherapy	
Yes	23 (6)
No	372 (94)
Adjuvant chemotherapy	
Yes	65 (16)
No	330 (84)
Adjuvant tamoxifen	
Yes	20 (5)
No	375 (95)
Metastases to 15 yrs	
Yes	155 (39)
No	238 (61)
Deaths to 15 yrs	
Dead	219 (55)
Alive	176 (45)

All blood samples were treated in an identical manner. Blood was allowed to clot, spun and the serum removed and aliquoted before storing at -20°C.

### Screening of sera on glycopeptide micro-arrays

Microarray slides were blocked in 50 mM ethanolamine in sodium borate buffer pH 8.5 at room temperature for one hour with gentle agitation. Slides were washed three times with PBS (phosphate buffered saline; 0.5M Na_2_HPO_4_, 0.15M NaCl, 0.3M KCl, 0.5M KH_2_PO_4_, pH 7.4), rinsed in MilliQ water and dried by centrifugation (Labnet, Oakland, Rutland, UK), C1303-T-230V-UK, 4,800 rpm). 25 ul (MPX16 well slides) or 5 uL (MPX48 well slides) of serum diluted (1:25 in 1 × PLI-P Buffer, 0.0065M Na_2_HPO_4_, 0.5M NaCl, 0.003M KCl, 0.0015M KH_2_PO4, 1% BSA, 1% Triton-X-100, pH 7.4) was added to each well and the slides incubated at room temperature for one hour with gentle agitation. Slides were washed with PBS wash buffer (PBS containing 0.05% Tween-20) and then with PBS, and then probed with Cy3 labelled anti-human IgG secondary antibody (1:1,000 dilution, C2571 Sigma, Gillingham, Dorset, UK) at room temperature, for one hour, with gentle agitation. Slides were then washed again and scanned with a single laser power and detector gain setting. The images were quantified with ProScanArray Express software program (PerkinElmer). Spots were identified using automated spot finding or manual adjustments for occasional irregularities. The spot intensities were determined by subtracting the median compound for each sample. The operator was blinded as to which samples were from breast cancer patients or controls and all samples were screened in duplicate with the same positive control serum from a breast cancer patient being run on every slide. The reproducibility of the assay was assessed by screening a subset of samples at two sites (KCL and University of Copenhagen). A total of 57 samples from cancer patients were assessed as positive in Copenhagen and 55 of these were positive in the screen at KCL indicating 96% agreement.

### Data analysis

#### Determination of positive sera

Each glycoform of MUC1 was synthesised on two overlapping peptides known as MUC1a and MUC1b (see above and Figure 1). The combination of MUC1a and MUC1b carrying one type of glycan is defined as the feature. Sera were defined as being positive if they reacted with one or both of the MUC1a and MUC1b glycopeptides that carried the same glycan, taking the maximum of the two values. A positive value was defined as being two standard deviations (SDs) above the mean of the reactivity of sera from patients with benign breast disease or healthy female controls, on the corresponding feature. A strong positive was defined as being four SDs above the means. These cut offs were chosen as the data are close to a half-normal distribution and it would, therefore, be expected that at least 95% of the data to be below +2 SDs. After sample analysis and image processing a data set was constructed. Chi-squared tests were used to determine the *P*-values in Tables 2 and 3.

**Table 2:** Sera reacting with MUC1 glycoforms when screened against arrays displaying glycoforms shown in Figure 1B

	Sera from patients with benign breast disease used to define cut-off		Sera from age-matched healthy females used to define cut-off	
	*P *< 0.0001		*P *= 0.0007	
	Breast cancer n (%)	Benign disease n (%)	Breast cancer n (%)	Healthy females n (%)
negative	271 (69)	97 (90)	272 (69)	85 (86)
positive^1^	124 (31)	11 (10)	123 (31)	14 (14)
strong positive^2^	97 (24)	8 (7)	80 (20)	7 (7)

^1^Sera was defined as positive if it was two standard deviations or higher than the mean fluorescence for at least one feature.

^2 ^Sera was defined as strong positive if four standard deviations or higher than the mean fluorescence for at least one feature

**Table 3:** Autoantibodies in cancer patient sera to MUC1 glycoforms correlate with age when sera were taken

		Sera from patients with benign breast disease used to define cut-off		Sera from age-matched healthy females used to define cut-off	
		All positives^1^	Strong positives^2^	All positives	Strong positives
		***P = 0.001***	***P = 0.001***	***P = 0.007***	***P = 0.003***
Age sera taken	N	n (%)	n (%)	n (%)	n (%)
<50	63^3^	7 (11)	3 (5)	14 (22)	5 (8)
50 to 59	132	33 (25)	27 (20)	31 (24)	21 (16)
60 to 69	136	57 (42)	46 (34)	51 (38)	39 (29)
70+	64	27 (42)	21 (33)	27 (42)	15 (23)

^1 ^Sera were defined as all positive if two standard deviations or higher than the mean fluorescence for at least one feature.

^2 ^Sera was defined as strong positive if four standard deviations or higher than the mean fluorescence for at least one feature.

^3 ^Total number in age group

#### ROC curve analysis

The sensitivity and specificity for every possible threshold decision rule was calculated to plot a receiving operational characteristic (ROC) curve for a single feature. Such a decision rule for a feature with value *x *would take the following form (in this example we will use benign vs malignant):

for a preselected threshold *θ*.

The generalised ROC was formed by finding a linear combination of features which gives the largest area under the ROC curve. The features, *x_i _*, for *i = 1,...,k *for *k *number features are combined into one `general' feature, *z*, by

where *w_1_,...,w_k _*are the weights for each feature which were set to 1 to reflect the fact that we did not have pre-disposition towards a particular feature. Also, we did not perform any model selection in order to fine-tune the best combination of weights: This was to avoid any data snooping inherent with such an approach.

#### Statistical tests used for clinical analyses

Comparisons of the distribution of positive status with clinical parameters were made using the Chi-squared test of significance. Comparisons of metastasis-free survival by positive status were made using the Kaplan Meier Log Rank test and Cox Proportional Hazards models were used for the multivariate analysis. These tests were performed using the SPSS (v.16) statistical package (IBM, Feltham, Middlesex, UK).

### Quantitative real-time-PCR

Frozen tissues (0.5 mm^3^) from 58 primary breast tumours, showing more than 70% tumour were crushed to powder using a Mikrodismembrator II (Sartorius) and lysed in Qiazol (Qiagen, Crawley, West Sussex, UK). Total RNA was extracted using the RNEasy Lipid tissue kit (Qiagen) according to manufacturer's instructions. The integrity of the extracted RNA was controlled using a Bioanalyser (Agilent, Wokingham, Berkshire, UK). DNased RNA (2 μg) was reverse transcribed using random hexamer primers and superscript *III *reverse transcriptase (Invitrogen, Paisley, Scotland, UK). QRT-PCR was performed using the Opticon qRT-PCR analysis system (Biorad, Hemel Hempstaed, Hertfordshire, UK), a hot-start PCR reaction that contained the ds-specific DNA-binding dye SYBR Green I (Sigma), and 100 pmol of the forward and reverse primers shown:

ACTB forward primer 5' CCCTCCATCGTCCACCGCAAATGCTTC 3':

ACTB reverse primer 5' CGACTGCTGTCACCTTCACCGTTCCAG 3'

B3GNT6 forward primer 5'CGGCTAGACTATCTCTTCATCCTC3'

B3GNT6 reverse primer 5'CCACTCACTTGTAAACAGTGGAAA3'

C1GALT1 forward primer 5'GAGGTGGCTTCTTTCAAAATACGACCC3'

C1GALT1 reverse primer 5'CATCTCCCCAGTGCTAAGTCTTCAATG3'

After 5 minutes at 95°C, 40 cycles were performed: 15 seconds denaturation at 94°C, 30 seconds annealing at 60°C, 30 seconds extension at 72°C and fluorescence detection at 78°C. A melting curve fluorescence analysis was performed on each sample once the amplification cycles were completed to verify that a single product had been amplified. Each sample was normalized to the housekeeping gene of β-actin (ACTB). All qPCR reactions were performed in triplicate.

## Results

### Sera from breast cancer patients contain autoantibodies to multiple MUC1 glycoforms

In our previous work screening for autoantibodies to MUC1 glycoforms, we used enzymatic solution synthesis of glycopeptides based on three tandem repeats of MUC1 and the sera were commercially obtained [13]. Using these arrays we confirmed that autoantibodies to MUC1 carrying the truncated O-linked glycans core3, STn - and to a lesser extent Tn - were indeed present in a proportion of the sera (54/198) from the early stage breast cancer patients to be analysed here (data not shown).

To examine the specificity of these antibodies in more detail, and to test if glycopeptides based on a single tandem repeat could be used for antibody detection, we used solid-phase glycopeptide synthesis with on-chip microarray purification [19] to construct a micro-array of glycopeptides based on a single 20 amino acid tandem repeat of MUC1 (MUC1a, see Figure 1). Thirty-one Tn MUC1 glycopeptides, carrying each glycan at different sites were chemically synthesized and extended on-chip with glycosyltransferases to form core3 and STn MUC1 glycopeptides (see Figure 2, [19] and Materials and methods).

Analysis with these arrays of five of the sera from early breast cancer patients found to be positive on the 60mer arrays confirmed that IgG autoantibodies could be detected to MUC1 carrying Tn, core3 and STn when using only a single tandem repeat. Figure 2 shows three examples of the profile of autoantibodies documented in the patients' sera. For clarity in presentation the reactivity with only seven informative MUC1 glycopeptides is shown. Autoantibodies could be identified in the sera from an individual patient that recognised several glycoforms carried on MUC1a, that is, Tn, STn and core3 (patient A), or that reacted with one particular glycoform (sera from patients B and C). Moreover, even though the same glycoform could be recognised by sera from patients A and B (core3MUC1) or from patients A and C (STnMUC1), the site carrying the glycan recognised by the antibodies was different. Therefore, the on-chip glycosylation is a rapid approach to reveal detailed autoantibody signatures to glycopeptides of MUC1.

### Screening of sera from a large cohort of early stage breast cancer patients, patients with benign breast disease, and healthy women

To produce sufficient quantities of glycopeptides required to screen large numbers of sera it was necessary to use a combination of chemical synthesis coupled with chain extension in solution with glycosyltransferases. To expedite the chemical synthesis of glycopeptides using Tn or core3 building blocks two MUC1 20mer glycopeptides (MUC1a and MUC1b) were designed with a 10mer overlap. MUC1a and MUC1b carrying Tn in three positions or core3 in two positions were chemically synthesized (see Figure 1B) and for technical reasons the glycopeptide carrying core3 at the PDTRP threonine position was synthesized separately. The TnMUC1 and core3MUC1 glycopeptides were enzymatically extended in solution to produce either the STn or ST glycoforms or the extended core3 structures respectively (see Table S1 in Additional file 1 for full list). These 20mer glycopeptides, and for comparison with the previous pilot study [13] a 60mer carrying core3, were printed onto slides and the arrays validated by the use of lectins and monoclonal antibodies (see Figure S1 in Additional file 2). The 60mer carrying core3 gave very similar results to the 20mer carrying core3 (see Figure S2 in Additional file 3). However, the use of the smaller glycopeptides allowed the synthesis of a wider selection of MUC1 glycoforms.

These arrays were used to screen 395 sera taken before surgery from breast cancer patients with Stage I or Stage II disease (P-series). Clinical parameters for these patients are listed in Table 1 and their average age was 59.9. The availability of extended clinical follow-up data allowed us to demonstrate that the cohort showed the expected correlations with the standard parameters for predicting prognosis (lymph node involvement, grade, tumour size and age at diagnosis, see Figure S3 in Additional file 4). Sera from 108 patients with benign breast disease who attended Guy's Hospital during a similar period were also analysed. In order to age-match the controls with the breast cancer patients, sera of age-ranged matched healthy females (*n *= 99, average age 54.7 years) who had not developed any form of cancer 25 to 30 years after donation were also included in the analysis.

**Figure 3:**
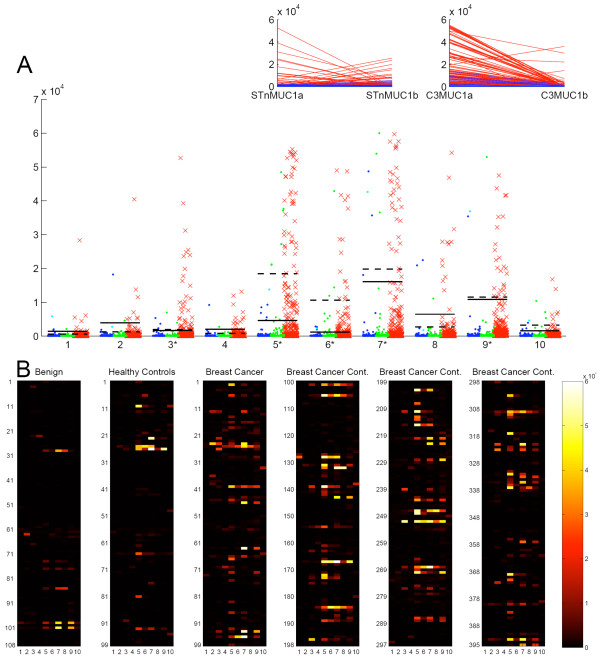
**Reactivity of auto-antibodies in sera with MUC1 glycoforms**. **A**, dot blot showing the reactivity of auto-antibodies present in sera from breast cancer patients, red crosses; patients with benign breast disease, blue circles; and healthy females aged ranged matched to the cancer patients, green circles. Bars indicate the position for 2x the standard deviation of the mean of the benign serum values, (solid bar) or the aged matched normal values, (dotted bar). Glycopeptides are defined in figure 1B. Inserts show the reactivity of autoantibodies in the sera of breast cancer patients with MUC1a and b carrying STn (left insert) and MUC1a and b carrying core3 (right insert). Each red line represents an individual serum sample. **B**, heat maps of the reactivity of autoantibodies in sera to glycopeptides 1 to 10 defined in figure 1B. Breast Cancer Cont, breast cancer sera continued.

The MUC1a and MUC1b glycopeptides giving informative results are listed in Figure 1B. Figure 3 shows the reactivity of the screened sera on the combined MUC1a and MUC1b individual glycan features both as a dot blot and a heat map, the latter allowing visualisation of the reactivity of each serum sample. The dot blot (Figure 3A) shows that the highest frequency of autoantibodies in the cancer patients' sera is to the core3 based and STn MUC1 glycopeptides and these also gave the strongest signals. Importantly, reactivity to the haptens core3 or STn linked to threonine, or to the unglycosylated MUC1 was weak and found in only a few patients thus demonstrating that the strongly binding autoantibodies were largely directed to epitopes determined by both the glycan and the core peptide.

The autoantibodies in breast cancer sera that reacted with STnMUC1 reacted either with the STnMUC1a or the STnMUC1b but not with both (see insert in Figure 3A). In addition, the majority of sera containing antibodies to core3 MUC1 showed reactivity with core3 MUC1a, with only four sera showing reactivity with MUC1b (see right insert in Figure 3A). Fewer and weaker responses were seen to ST and Tn MUC1.

### Expression of β3GnT6 by breast carcinomas

The core3 structure is synthesized by the enzyme core3 β3-N-acetylglucosamine transferase which is encoded by the β3GNT6 gene. This glycosyltransferase is not expressed in the normal mammary gland and it has not been found in breast carcinomas [23]. We, therefore, looked by RT-qPCR at the expression of β3GNT6 in 58 Stage 1 and Stage 2 breast carcinomas (see Figure S4 in Additional file 5). As expected, normal colon showed reasonable levels of the transcript. However, in only 3 out of 58 breast carcinoma samples was a β3GNT6 transcript detectable and these were at least 100-fold lower than the glycosyltransferase, C1GALT1, that competes for the same sugar substrate to form core 1 (T). This observation together with published data, presents compelling evidence that core3 is not expressed by breast carcinomas suggesting that the MUC1-core3 glycan on the microarray may be acting as an epitope mimic.

### Quantitative analysis of the screening assay to define serum reactivity

Table 2 shows the number and percentage of positive (defined as two standard deviations above the means) and strongly positive (defined as four standard deviation above the means) with one or more of the glycopeptides described in Figure 1B for the breast cancer patients, patients with benign breast disease and healthy controls, using either the benign sera or the sera from healthy females to define the cut offs.

Thirty-one percent of the breast cancer patients had IgG autoantibodies to the glycopeptides whether the sera from patients with benign breast disease or sera from healthy women were used as controls. Both control cohorts showed a 7% rate of strongly positive sera, and using the cut off from data obtained from sera from patients with benign disease, 24% of the cancer patient sera were strongly positive. This was reduced to 20% when the sera from healthy females (who did not develop cancer) were used for establishing the cut off. We found a highly significant correlation of the presence of antibodies with age in the cancer patients, which was observed whether the cut-off used was from the benign or the more closely aged matched controls (see Table 3). Therefore this difference of 20% versus 24% may be due the difference in age of the control cohorts.

**Figure 4:**
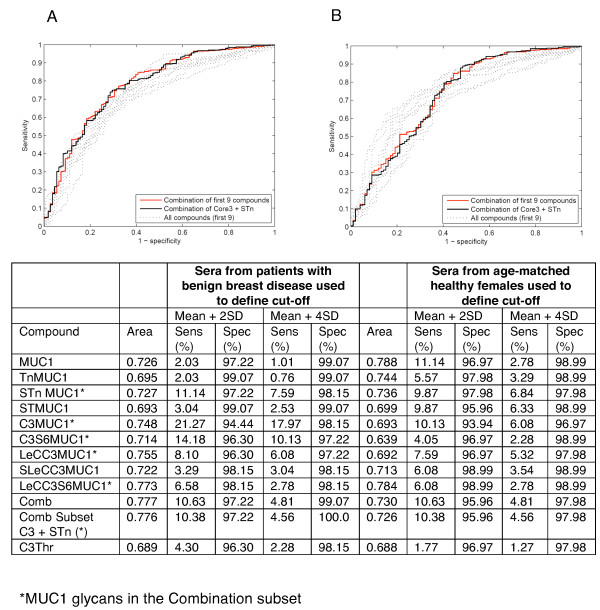
**Receiver operating characteristic analysis of individual and combinations of features**. **A**, sera from patients with benign breast disease defines cut-offs; **B**, sera from healthy females defines cut-offs. Pale dotted grey lines represent individual features, solid red line represent the combination of all the features and solid black line represents the combinational subset defined in Table by * and Figure 1B by *. The table shows ROC curve area and sensitivity/specificity data for breast cancer sera on each MUC1 glycan, combination of all MUC1 glycans and a combination subset.

ROC curves, shown in Figure 4, were constructed for each of the nine features (see 'Materials and methods') using either the benign serum values to provide the cut off or the values from the age matched healthy individuals. Giving an equal weight to all features (shown in pale grey lines) we formed a combination (generalised ROC curve) to give a single curve (red line). Figure 4 shows that this gave an area under the curve of 0.777 and 0.730 when using the benign sera or sera from healthy females to define cut-offs respectively. Using sera from benign patients to define cut-offs, the generalised ROC curve showed an improvement over the curves of the individual features. When looking at the dot plots in Figure 3A it is obvious that some glycoforms contribute more information and the STn and core3 glycoforms give both high positive breast cancers along with a low positive benign. Using these criteria, we constructed a ROC curve of this subset of glycoforms (combinational subset defined in Figure 1B) and this is shown by the solid black line in Figure 4. The area under this ROC curve is close to the ROC curve area for the combination of all features. When the generalised and combinational ROC curves were applied to the samples using sera from the healthy women as the controls these curves were not as informative as some of the individual features (see Figure 4B) but in these latter cases, only a small number of sera were positive (see Figure 3A).

### Prognostic significance of autoantibodies to MUC1 glycoforms

To investigate correlations with clinical parameters we used the healthy female control sera to define the cut-offs as they were more closely age matched. We used two standard deviations above the mean for each glycopeptide to define as all positives and four standard deviations above the mean to define strong positives. The presence of IgG autoantibodies was then compared to a series of clinical parameters including occurrence of metastases within 15 years, grade, lymph node involvement, tumour size, ER positivity, parity and age. When IgG autoantibodies to all the glycoforms screened were analysed there was a correlation with tumour grade but no other parameters (data not shown). However, using the subset defined in Figure 1B and Figure 4, Table 4 shows that the highly significant correlation with age remained (*P *= 0.001) and a significant correlation was observed with tumour grade when the strong positives were analysed (*P *= 0.016). Moreover, autoantibodies to this combinational subset were associated with a lower incidence of metastasis with a follow-up of 15 years (see Table 4).

**Table 4:** Correlation of sera autoantibodies to MUC1 glycopeptide combinational subset with clinical variables

		Autoantibody positive		Strong autoantibody positive^A^
	N^B^	% positive	N^A,B^	% positive
Age at diagnosis		***P = 0.001^C^***		***P < 0.001***
<50	63	5	61	2
50 to 59	132	14	125	9
60 to 69	136	27	129	23
70+	64	27	57	18
Stage		*P = 0.215*		*P = 0.104*
Node negative	200	22	189	17
Node positive	193	17	181	11
Positive nodes		*P = 0.195*		*P = 0.219*
0	200	22	189	17
1 to 3	124	19	114	12
4+	69	12	67	9
Clin tumour size		*P = 0.483*		*P = 0.306*
0 to 2 cm	133	19	129	16
2.1 to 4 cm	176	20	162	13
>4 cm	74	14	70	9
Tumour grade/type		*P = 0.165*		***P = 0.016***
ID Grade I	37	30	35	26
ID Grade II + I Lob	195	19	187	15
ID Grade III	120	16	109	7
ER Status		*P = 0.883*		*P = 0.183*
ER negative	131	18	120	11
ER positive	211	19	204	16
Parity		*P = 0.730*		*P = 0.436*
0 children	87	23	82	18
1 to 2 children	171	19	164	16
3+ children	96	19	88	11
Metastases to 15 yrs		*P = 0.058*		***P = 0.028***
No	238	22	237	17
Yes	155	14	133	9

^A ^Weak positives excluded

^B ^Total number in that group for the variable.

^C ^Using Chi-squared test for equality of distributions Bold text indicates a significant correlation.

**Figure 5:**
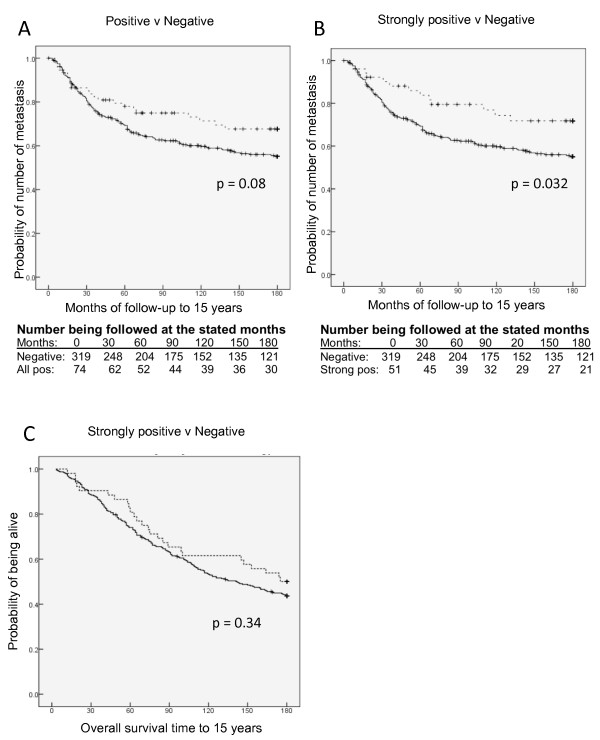
**Breast cancer patients with autoantibodies to MUC1 glycopeptide combinational subset show increased time to metastasis**. **A**, metastasis free survival with patients with autoantibodies versus no auto-antibodies (*P *= 0.08). **B**, metastasis free survival of patients with strong autoantibodies versus no antibodies (*P *= 0.032). **C**, overall survival of patients with strong autoantibodies versus no antibodies. Thick lines indicate no auto-antibodies in sera. Dotted lines indicate autoantibodies. Vertical dashes indicate censored events.

The metastasis-free survival was plotted for the sera containing IgG autoantibodies to glycopeptides in the combinational subset compared to negative sera. Figure 5 shows that there was a separation of the curves and Log Rank testing gave a *P*-value of 0.081 for all positives and 0.032 for the strong positives. However, when using Cox regression analysis adjusted for age, nodal status, grade and tumour size, this association was lost (*P *= 0.834, data not shown). This suggests that the presence of autoantibodies is not an independent variable but rather reflects the clinical condition of a patient with early disease. There was also no significant association of strongly positive sera with overall survival to 15 years in either univariate, see Figure 5C, or multivariate analysis (*P *= 0.601, data not shown).

## Discussion

The efforts to diagnose cancer by the use of serum assays have met with limited success, partly because the disease is heterogeneous at both causative and host response levels. The changes in O-linked glycosylation that occur in cancer [16,23] result in the production of cancer-associated glycoforms of cancer-associated glycoproteins, which are antigenically distinct and are able to induce IgG autoantibodies [13,24,25]. By taking into account these modifications, the specificity of diagnostic markers might be expected to be improved, as they reflect both the patho-physiology of the disease and the hosts' response. Using novel micro-arrays carrying different glycoforms of MUC1 we have demonstrated the presence of autoantibodies in the sera of a proportion of breast cancer patients that recognise MUC1 carrying specific O-linked glycans. The difference in the percentage of sera strongly positive for the antibodies in cancer patients' sera versus sera from patients with benign disease or from healthy individuals who never developed cancer is highly significant.

Unglycosylated MUC1, or undefined glycoforms of MUC1, has been used in other ELISA based assays for detecting antibodies in sera from cancer patients [1-3,12]. However, using the microarray assay described here, the antibody response to un-glycosylated MUC1 was very weak and only seen in a very small number of patients (see Figure 3A). In contrast, IgG autoantibodies to defined MUC1 glycoforms were found in 31% of Stage I and II breast cancer patients. Importantly, elevated levels of circulating MUC1 antigen (CA15-3) was not detected in these serum samples from early stage patients. In contrast, in late stage patients, where MUC1 antigen can be detected in sera, IgG autoantibodies were not detected (data not shown). This emphasises the importance of the autoantibody assay in early breast cancer when the antigen itself cannot be detected and the induction of autoantibodies represents an amplification of the antigenic signal.

Autoantibodies were detected to MUC1 carrying core3 based glycans and STn, with fewer responses seen to Tn and ST glycoforms. The STn glycan is relatively tumour specific, but expression of this glycan is seen in only 25 to 30% of breast cancers, which could explain the lower percentage of sera showing IgG autoantibodies to STnMUC1 compared to other cancers such as colo-rectal [13,19]. Although the ST glycoform is the main MUC1 glycoform found in sera of advanced breast cancer patients [26], fewer and weaker responses were seen to STMUC1. ST is also found on other serum proteins, therefore, tolerance might be expected to be operative.

The dominance of antibodies to core3MUC1 in the breast cancer sera is surprising. The enzyme catalysing the addition of GlcNAc to GalNAc to produce core3 (β3GNT6) is generally only expressed in the cells of the gastrointestinal (GI) tract and it is dramatically downregulated in colon cancer [27,28]. We found that the β3GNT6 transcript could only be detected in 3 of 58 breast carcinomas, and even then the level was at least 100-fold lower than the competing glycosyltransferase, C1GALT1. Moreover, core3 structures have not been found in normal breast or breast carcinomas [23]. While clearly the autoantibodies recognise core-3 structures on MUC1 (but not the core3-threonine), it is possible that the antibodies were induced to a structure that is mimicked by MUC1 carrying core-3.

Our data (Table 3) show that the presence of autoantibodies in the breast cancer patients is highly correlated with age, emphasising the importance of using aged-matched controls for any autoantibody study. The increase of autoantibodies with age has been reported for both mice and man [29]. Also, in a study by Pinheiro *et al.*, measuring antibodies to unglycosylated MUC1 by ELISA in sera collected in the Nurses Health Studies, an association with age was noted in women with antibodies who went on to develop ovarian cancer [12]. The increased specificity and sensitivity of the assay when using sera from patients with benign breast disease to determine cut-offs almost certainly reflects the younger age of the patients with benign disease. However, the correlation with age that we observed was maintained when the data were analysed using sera from the age range matched healthy females.

The availability of ideal control sera for use in retrospective studies can be a problem. The sera from patients with benign breast disease were collected at the same site and at around the same time as the cancer patients, thus reflecting the same population for both cohorts. However, the age demographics of benign breast disease results in the mean age of these patients being significantly younger than the breast cancer patients. The healthy control sera used were taken in the 1970s from healthy females living on the island of Guernsey [22] and allowed not only closer age matching, but extensive follow-up, although the ethnicity of the Guernsey and P-series populations may not be identical. Nevertheless, the availability of extensive follow-up allowed for the selection of sera from Guernsey women who had not developed any form of cancer for 25 to 30 years after blood donation. It should be noted that sera from all three cohorts were stored under identical conditions and for similar periods of time. Using sera from healthy women to determine cut-off points we found a significant association of the presence of autoantibodies to the combinational subset with increased time to metastasis (see Figure 5). Although this was not an independent variable, it should be noted that when the presence of autoantibodies to any of the other glycopeptides screened were analysed, no such significant association was observed (data not shown). This suggests that autoantibodies to the combinational subset could play a role in disease progression.

An important question which should be addressed is how long before clinical symptoms develop can IgG autoantibodies to aberrant glycoforms of MUC1 be detected? We have anecdotal evidence that the antibodies may appear early since one of the patients who had benign disease and high levels of IgG autoantibodies to MUC1 glycopeptides, developed breast cancer five years later. However, antibodies to the subset of MUC1 glycopeptides were found in a number of the control sera taken from the cohort of women on Guernsey where no cancer developed during 25 to 30 years of follow-up. The possibility arises that these antibodies may be functional in suppressing tumour development, as well as progression. This may be particularly true for the anti-STn-MUC antibodies (found in 2% of controls) as this glycoform is truly cancer specific and therefore the presence of antibodies should indicate the presence of tumour, albeit on a micro scale. However, as MUC1 and core3 are expressed in the colon there is the possibility that antibodies to MUC1core3 may be induced during an inflammatory response occurring in the colon resulting in expression of shorter glycans [30]. However, following up individuals who have antibodies would not be an overwhelming procedure as it merely involves taking blood and screening with the arrays for antibody levels- possibly in parallel with mammography screening.

In searching for a simple, cost effective and non-invasive screening assay that could pick up breast malignancy in its early stages it should be noted that the MUC1 mucin is expressed by other carcinomas such as ovarian and lung, and IgG autoantibodies to MUC1 carrying core3 or STn glycans have been detected in sera from patients with these cancers [13]. Thus, an assay based solely on aberrant glycoforms of MUC1 applied to sera from individuals without clinical symptoms would not necessarily distinguish between the different carcinomas. To extend this work we are using our on-chip technology and other glycopeptide platforms for high-throughput screening and discovery of disease-associated autoantibody glycopeptide epitopes in other proteins with more organ specific expression patterns.

## Conclusions

Whereas the serum assay (CA15.3) used for detecting the cancer associated MUC1 antigen in breast cancer patient serum does not detect elevated antigen in early stage breast cancer, autoantibodies to specific tumour-associated glycoforms of MUC1 can be detected in sera from Stage I and Stage II breast cancer patients. The difference in the percentage of sera strongly positive for the antibodies in cancer patient sera versus sera from patients with benign disease or from healthy individuals who never developed cancer is highly significant. Follow-up of patients with benign breast disease with sera with high levels of autoantibodies may be appropriate. The lower incidence of metastases and delay in development of metastases in patients with high levels of antibodies to a subset of the glycoforms, suggests that the antibodies may play a role in inhibiting the progression of disease. Administration of antibodies with these specificities is, therefore, a possible therapeutic strategy. This would be particularly appropriate for breast cancer patients with STn positive cancers (25 to 30% of breast cancer patients) as the STnMUC1 glycoform is truly tumour specific.

## Abbreviations

ACTB: β-actin; Gal: galactose; GI: gastrointestinal: PB: print buffer; PBS, phosphate buffered saline; ROC: receiving operational characteristic; SDs: standard deviations.

## Competing interests

OB has shares in GlycoZym, Inc. and is a consultant for GlycoZym, Inc. The other authors declare that they have no competing interests.

## Authors' contributions

OB contributed to the strategic plan and design of the experiments, developed the array assay and on-chip glycosylation methods, and contributed to the writing of the manuscript. DB performed most of the screening for antibodies in the sera of the large cohorts of breast cancer patients, patients with benign disease and healthy adults. BB performed the statistical analysis of the data obtained from the screening assays. DA selected the sera from the healthy adults, provided the clinical data for the breast cancer patients, and analysed the relation between clinical parameters and presence of antibodies. SJ analysed the levels of the core3 and C1GalT transferases in breast cancers and colon. MH contributed to the strategic plan of the work. AG oversaw the statistical analysis of the data generated. IF provided the follow-up data for the healthy controls and contributed to the design of the experiments. JTP contributed to the strategic plan and design of the experiments, and to the writing of the first draft and subsequent drafts of the manuscript. JB contributed to the strategic plan and design of the experiments, and to the writing of the first draft and subsequent drafts of the manuscript. All authors read and approved the final manuscript

## Supplementary Material

Additional file 1 **Supplementary Table 1**. Description of MUC1 glycoforms printed onto the slides. Table describing the glycopeptides printed onto the microarrays used to screen the large cohorts. Click here for file

Additional file 2 **Supplementary Figure 1**. Lectin and mAb binding to 48-well 20mer glycoform array. Staining of arrays with glycoform-specific lectins and antibodies for glycopeptide array quality control. Click here for file

Additional file 3 **Supplementary Figure 2**. Comparison of binding to 20mer MUC1 core3 and 60mer core3 of autoantibodies. Binding to MUC1 20mer (1TR) and MUC1 60mer (3TR) of individual sera. Click here for file

Additional file 4 **Supplementary Figure 3**. Clinical characteristics of the 395 breast cancer patients who donated sera for the study. Time to metastasis of the breast cancer cohort related to clinical tumour size, lymph nodes positivity, age at diagnosis and tumour grade. Click here for file

Additional file 5 **Supplementary Figure 4**. Expression of B3GNT6 in breast cancers. qRT-PCR of B3GNT6 in 58 primary breast cancers. Click here for file
