# Supplementary material for: Autoantibodies to aberrantly glycosylated MUC1 in early stage breast cancer are associated with a better prognosis
Source: Breast Cancer Res. 2011 Mar 8;13(2):R25. doi: 10.1186/bcr2841 (PMC3219186; doi:10.1186/bcr2841)
Supplement: Additional file 1 — Supplementary Table 1. Description of MUC1 glycoforms printed onto the slides. Table describing the glycopeptides printed onto the microarrays used to screen the large cohorts. [file bcr2841-S1.PDF]

Supplementary Table 1: Description of MUC1 glycoforms printed on to the slides

No on graph	Abbreviation	Sequence	Glycoform*
1	MUC1a	VTSAPDTRPAPGSTAPPAGH	N/A
2	MUC1b	APGSTAPPAHGVTSAPDTRP	N/A
3	TnMUC1a	VTSAPDT*RPAPGS*T*APPAGH	GalNAcα-
4	TnMUC1b	APGSTAPPAHGVT*S*APDT*RP	GalNAcα-
5	STnMUC1a	VTSAPDT*RPAPGS*T*APPAGH	Neu5Acα2-6GalNAcα-
6	STnMUC1b	APGSTAPPAHGVT*S*APDT*RP	Neu5Acα2-6GalNAcα-
7	TMUC1a	VTSAPDT*RPAPGS*T*APPAGH	Galβ1-3GalNAcα-
8	TMUM1b	APGSTAPPAHGVT*S*APDT*RP	Galβ1-3GalNAcα-
9	3STMUC1a	VTSAPDT*RPAPGS*T*APPAGH	Neu5Acα2-3Galβ1-3GalNAcα-
10	3STMUC1b	APGSTAPPAHGVT*S*APDT*RP	Neu5Acα2-3Galβ1-3GalNAcα-
11	C3MUC1a	VTSAPDTRPAPGS*T*APPAGH	GlcNAcβ1-3GalNAcα-
12	C3MUC1b	APGSTAPPAHGVT*S*APDTRP	GlcNAcβ1-3GalNAcα-
13	C3S6MUC1a	VTSAPDTRPAPGS*T*APPAGH	GlcNAcβ1-3[Neu5Acα2-6]GalNAcα-
14	C3S6MUC1b	APGSTAPPAHGVT*S*APDTRP	GlcNAcβ1-3[Neu5Acα2-6]GalNAcα-
15	LeCC3MUC1a	VTSAPDTRPAPGS*T*APPAGH	Galβ1-3GlcNAcβ1-3GalNAcα-
16	LeCC3MUC1b	APGSTAPPAHGVT*S*APDTRP	Galβ1-3GlcNAcβ1-3GalNAcα-
17	SLeCC3MUC1a	VTSAPDTRPAPGS*T*APPAGH	Neu5Acα2-3Galβ1-3GlcNAcβ1-3GalNAcα-
18	SLeCC3MUC1b	APGSTAPPAHGVT*S*APDTRP	Neu5Acα2-3Galβ1-3GlcNAcβ1-3GalNAcα-
19	LeCC3S6MUC1a	VTSAPDTRPAPGS*T*APPAGH	Galβ1-3GlcNAcβ1-3 [Neu5Acα2-6]GalNAcα-
20	LeCC3S6MUC1b	APGSTAPPAHGVT*S*APDTRP	Galβ1-3GlcNAcβ1-3 [Neu5Acα2-6]GalNAcα-
21	C3MUC1aPDTR	VTSAPDT*RPAPGSTAPPAGH	GlcNAcβ1-3GalNAcα-
22	C3MUC1-60mer	[VT*SAPDTRPAPGS*T*APPAGH]$_3$	GlcNAcβ1-3GalNAcα-
23	REC-MUC1		N/A
24	REC-TnMUC1		GalNAcα-
25	REC-TMUC1		Galβ1-3GalNAcα-
26	REC-STMUC1		Neu5Acα2-3Galβ1-3GalNAcα-
27	C3-Threonine		GlcNAcβ1-3GalNAcα-Thr
28	STn- Threonine		Neu5Acα2-6GalNAcα–Thr

* Indicates sites of glycosylation

Rec- Indicates recombinant full length MUC1 produced in CHO cells (REC-TMUC1 and REC-STMUC1 or ldlD CHO cells (REC-MUC1 and REC-TnMUC1)
