# Supplementary material for: Autoantibodies to aberrantly glycosylated MUC1 in early stage breast cancer are associated with a better prognosis
Source: Breast Cancer Res. 2011 Mar 8;13(2):R25. doi: 10.1186/bcr2841 (PMC3219186; doi:10.1186/bcr2841)
Supplement: Additional file 2 — Supplementary Figure 1. Lectin and mAb binding to 48-well 20mer glycoform array. Staining of arrays with glycoform-specific lectins and antibodies for glycopeptide array quality control. [file bcr2841-S2.PDF]

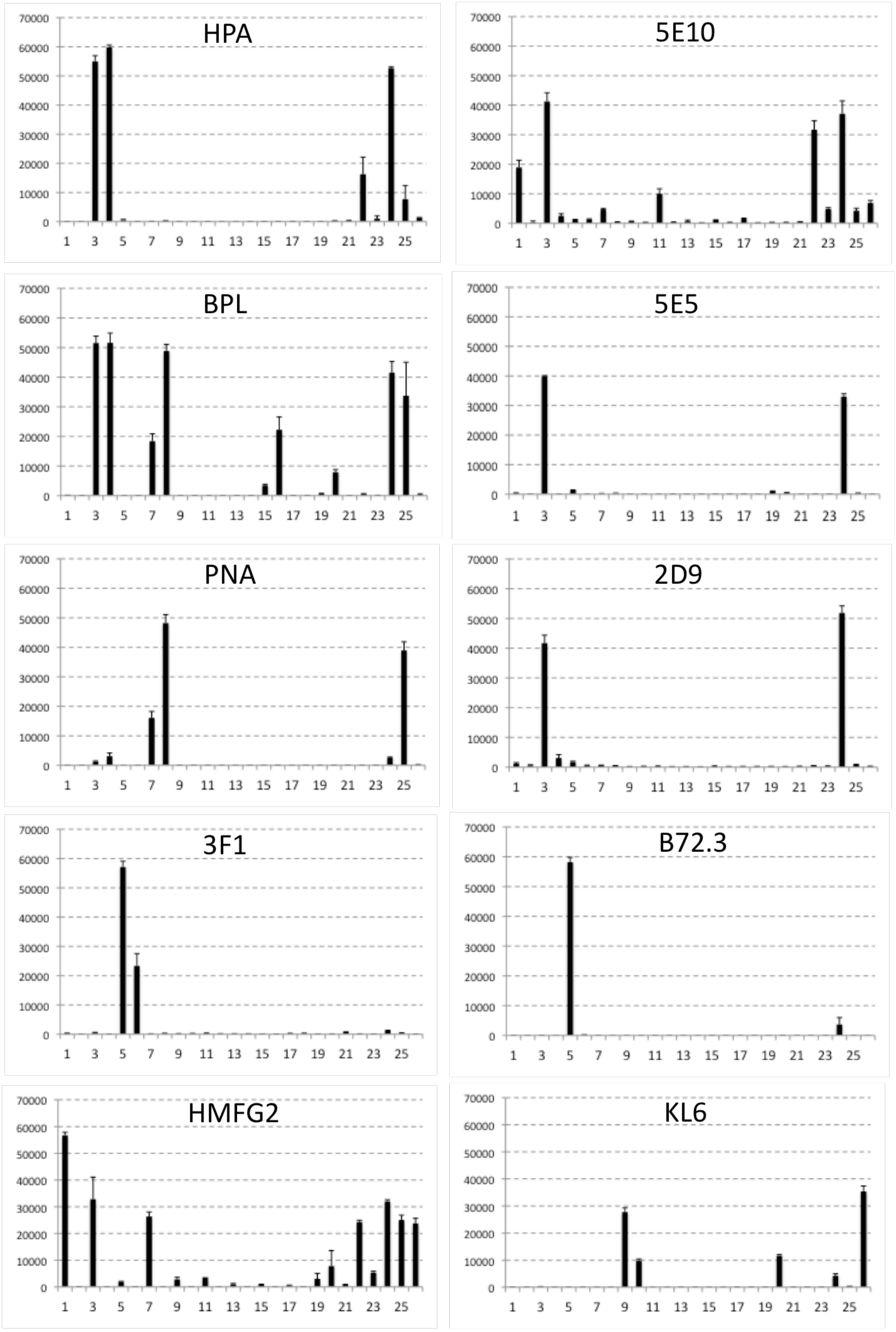

Supplementary Figure 1: Lectin and mAb binding to 48-well 20mer glycoform array (see supplementary table 1 for description of glycopeptides). Biotinylated lectins HPA (Sigma), BPL (Vector labs) and PNA (Vector labs) were used at 1ug/mL followed by detection with Streptavidin-Cy3 (Sigma) (1:1000 dilution) using incubation buffer (Material and Methods). Monoclonal antibodies were analyzed at 1ug/mL and detected with anti-mouse IgG –Cy3 labeled secondary antibody (1:1000 dilution).

HPA, specifically binds glycopeptides containing Tn, PNA lectin recognises T glycoforms and BPL recognises both Tn and T glycoforms. 3F1 recognizes any STn- glycoforms whereas mAb B72.3 specifically binds to STn-MUC1a glycopeptide. mAbs HMFG2 binds MUC1a peptides as well as Tn, T and Core-3 MUC1a glycopeptides but not STn-MUC1a. The mAb 5E5, 5E10 and 2D9 are specific to Tn-MUC1a peptides [17,18] and bind to such structures. The KL6 mAb is specific to the ST-MUC1 glycopeptides which is in agreement with recent studies [31].
