# Supplementary material for: Autoantibodies to aberrantly glycosylated MUC1 in early stage breast cancer are associated with a better prognosis
Source: Breast Cancer Res. 2011 Mar 8;13(2):R25. doi: 10.1186/bcr2841 (PMC3219186; doi:10.1186/bcr2841)
Supplement: Additional file 3 — Supplementary Figure 2. Comparison of binding to 20mer MUC1 core3 and 60mer core3 of autoantibodies. Binding to MUC1 20mer (1TR) and MUC1 60mer (3TR) of individual sera. [file bcr2841-S3.PDF]

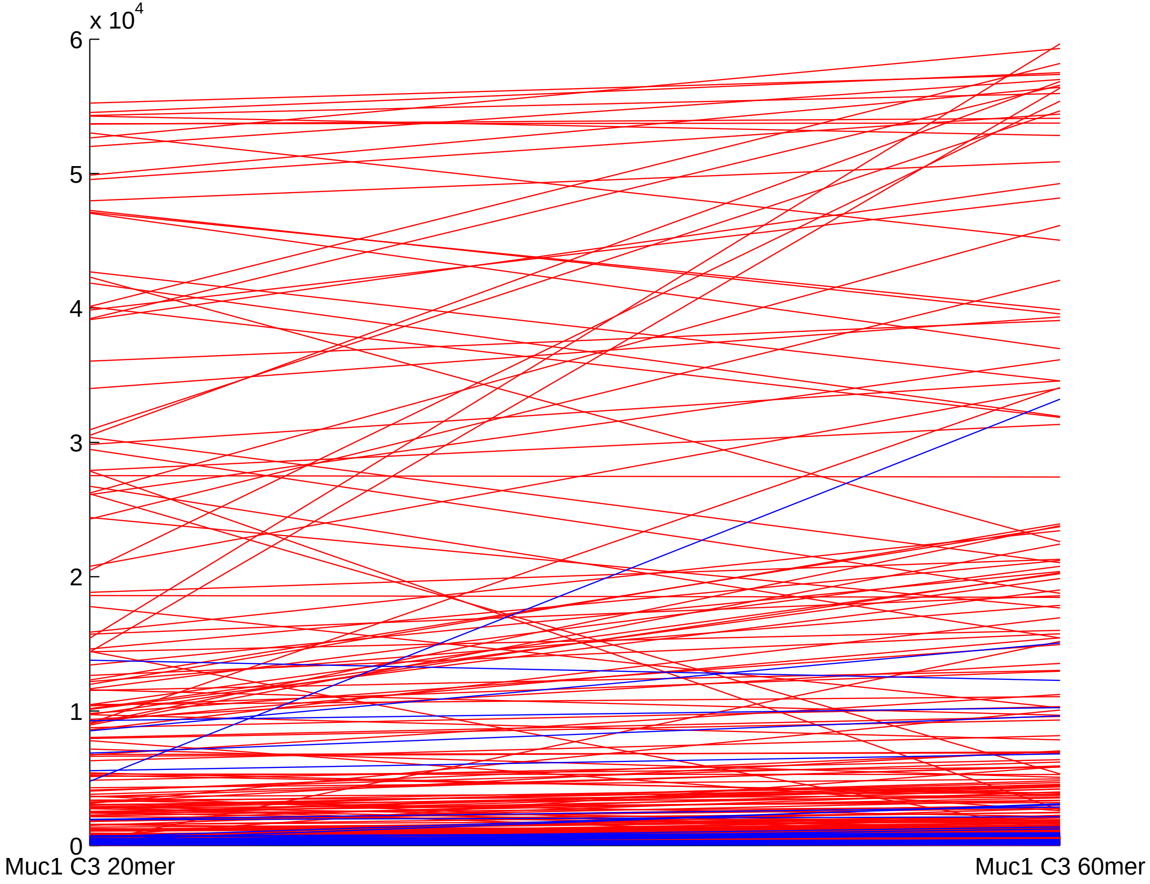

Supplementry Figure 2: Comparison of the binding to 20mer MUC1 core3 and 60mer MUC1 core3 of auto-antibodies sera. Each line represents an individual serum sample (red sera from breast cancer patients, blue healthy controls) binding to MUC1 20mer core3 (left hand side) and MUC1 60mer core 3 (right hand side).
