# Supplementary material for: Autoantibodies to aberrantly glycosylated MUC1 in early stage breast cancer are associated with a better prognosis
Source: Breast Cancer Res. 2011 Mar 8;13(2):R25. doi: 10.1186/bcr2841 (PMC3219186; doi:10.1186/bcr2841)
Supplement: Additional file 4 — Supplementary Figure 3. Clinical characteristics of the 395 breast cancer patients who donated sera for the study. Time to metastasis of the breast cancer cohort related to clinical tumour size, lymph nodes positivity, age at diagnosis and tumour grade. [file bcr2841-S4.PDF]

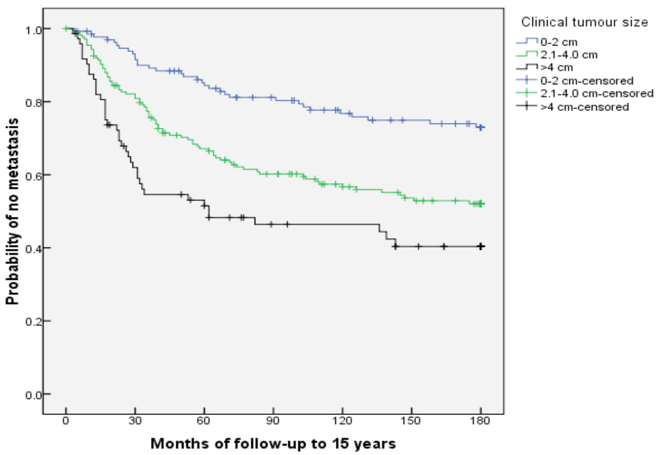


**Number being followed at the stated months**

| Months: | 0 | 30 | 60 | 90 | 120 | 150 | 180 |
|---|---|---|---|---|---|---|---|
| 0 – 2 cm: | 133 | 118 | 105 | 95 | 84 | 77 | 70 |
| 2.1 – 4 cm: | 175 | 138 | 109 | 93 | 77 | 69 | 59 |
| >4 cm: | 73 | 42 | 32 | 24 | 23 | 18 | 14 |

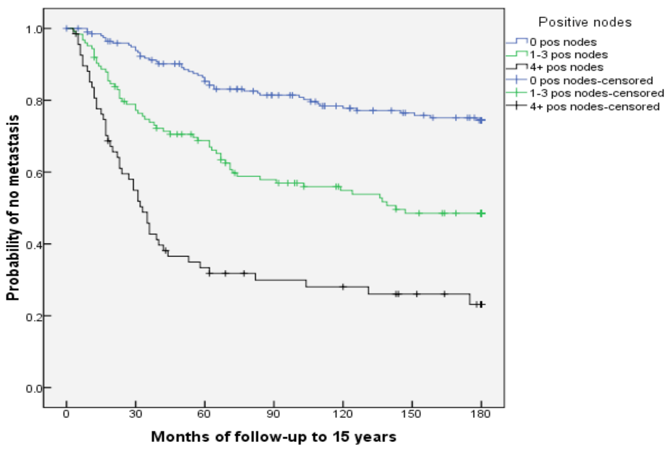


**Number being followed at the stated months**

| Months: | 0 | 30 | 60 | 90 | 120 | 150 | 180 |
|---|---|---|---|---|---|---|---|
| 0 nodes: | 199 | 180 | 157 | 140 | 124 | 114 | 103 |
| 1-3 nodes: | 124 | 93 | 77 | 62 | 52 | 45 | 40 |
| 4+ nodes: | 68 | 36 | 21 | 16 | 14 | 11 | 6 |

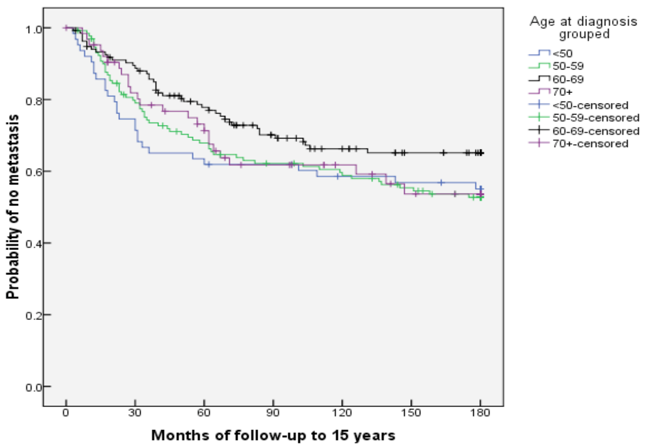


**Number being followed at the stated months**

| Months: | 0 | 30 | 60 | 90 | 120 | 150 | 180 |
|---|---|---|---|---|---|---|---|
| <50: | 63 | 45 | 39 | 34 | 34 | 33 | 30 |
| 50-59: | 131 | 100 | 84 | 75 | 70 | 64 | 55 |
| 60-69: | 135 | 117 | 95 | 76 | 63 | 55 | 48 |
| 70+: | 64 | 48 | 38 | 30 | 24 | 19 | 16 |

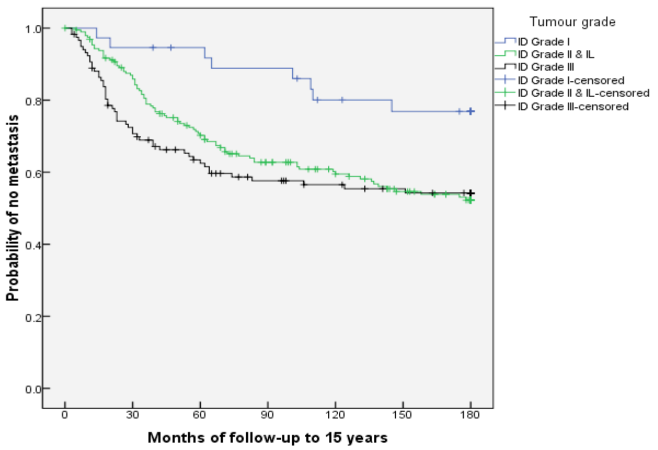


**Number being followed at the stated months**

| Months: | 0 | 30 | 60 | 90 | 120 | 150 | 180 |
|---|---|---|---|---|---|---|---|
| ID Grade I: | 37 | 35 | 33 | 31 | 26 | 24 | 22 |
| Grade II&IL: | 195 | 161 | 126 | 103 | 88 | 74 | 61 |
| ID Grade III: | 118 | 81 | 66 | 56 | 51 | 47 | 43 |

**Supplementary Figure 3: Clinical characteristics of the 395 breast cancer patients who donated sera for the study**
