# Supplementary material for: Autoantibodies to aberrantly glycosylated MUC1 in early stage breast cancer are associated with a better prognosis
Source: Breast Cancer Res. 2011 Mar 8;13(2):R25. doi: 10.1186/bcr2841 (PMC3219186; doi:10.1186/bcr2841)
Supplement: Additional file 5 — Supplementary Figure 4. Expression of B3GNT6 in breast cancers. qRT-PCR of B3GNT6 in 58 primary breast cancers. [file bcr2841-S5.PDF]

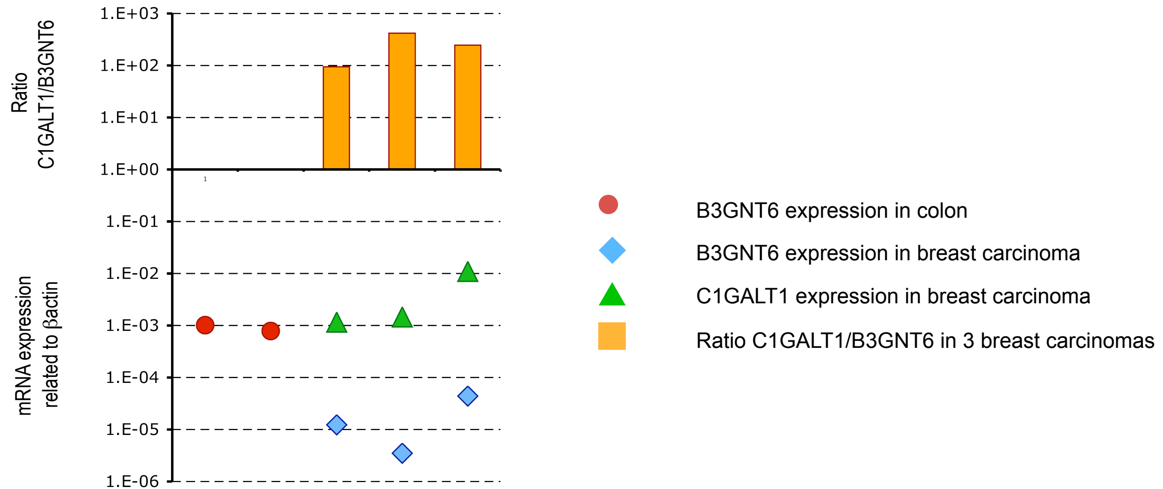

Supplementary figure 4: Expression of B3GNT6 in breast cancers. B3GNT6 transcript could only be detected in 3 of the 58 breast carcinomas analysed.
